# Petrochemical Equipment Tracking by Improved Yolov7 Network and Hybrid Matching in Moving Scenes

**DOI:** 10.3390/s23094546

**Published:** 2023-05-07

**Authors:** Zhenqiang Wei, Shaohua Dong, Xuchu Wang

**Affiliations:** 1 College of Safety and Ocean Engineering, China University of Petroleum, Beijing 102249, China; 2 CNPC Research Institute of Safety & Environment Technology, Beijing 102206, China; 3 Key Laboratory of Optoelectronic Technology and Systems of Ministry of Education, Chongqing University, Chongqing 400040, China; 4 College of Optoelectronic Engineering, Chongqing University, Chongqing 400040, China

**Keywords:** petrochemical equipment track, Yolov7 object detection, hybrid matching, attention mechanism, convolutional neural networks

## Abstract

Petrochemical equipment tracking is a fundamental and important technology in petrochemical industry security monitoring, equipment working risk analysis, and other applications. In complex scenes where the multiple pipelines present different directions and many kinds of equipment have huge scale and shape variation in seriously mutual occlusions captured by moving cameras, the accuracy and speed of petrochemical equipment tracking would be limited because of the false and missed tracking of equipment with extreme sizes and severe occlusion, due to image quality, equipment scale, light, and other factors. In this paper, a new multiple petrochemical equipment tracking method is proposed by combining an improved Yolov7 network with attention mechanism and small target perceive layer and a hybrid matching that incorporates deep feature and traditional texture and location feature. The model incorporates the advantages of channel and spatial attention module into the improved Yolov7 detector and Siamese neural network for similarity matching. The proposed model is validated on the self-built petrochemical equipment video data set and the experimental results show it achieves a competitive performance in comparison with the related state-of-the-art tracking algorithms.

## 1. Introduction

Automatic object tracking and analysis in industrial scenes is an important topic of computer vision technology that is receiving increasing attention along with the progress of intelligent industry 4.0 planning. Petrochemical equipment is the key target of on-site inspection and maintenance in petrochemical industry. Using mobile imaging device to track key refining equipment in petrochemical enterprises can greatly improve the automation level in many inspection and maintenance operations, such as automatic target positioning, automatic working status analysis, etc. Therefore, the research of automatic target tracking technology in mobile camera viewpoint has important theoretical value and huge application prospects.

In view of the shape characteristics of petrochemical equipment, most traditional tracking algorithms use probability density and image edge features as tracking standards, and take the rising direction of probability gradient as the target search direction. Although these algorithms are easy to deploy, their feature representation performance is poor, and they cannot handle target tracking in complex scenes, where the multiple pipelines present different directions and many kinds of equipment have huge scale and shape variation in serious mutual occlusions. The combination of deep learning and image processing can improve the performance of feature extraction and the processing speed is much faster than that of traditional algorithms. Therefore, the visual tracking strategies based on depth learning technique have presented greater advantages in performance. Among them, the “tracking-by-detection” approach that first detect objects and then tracks them is one of the most widely used tracking algorithms.

Currently, the “tracking-by-detection” approach can be roughly divided in two categories: SDE-based method that separately learns the detector and embedding for tracking [1,2,3,4] and JDE-based method that jointly learns the detector and embedding for tracking [5,6]. The SDE-based method mainly consists of three steps. Firstly, multiple possible targets in a single frame are detected by a target detection module, and their class and position are extracted. Secondly, these kinds of information are input into a tracking module, and the appearance features of the target are extracted. Finally, the target detection results and tracking results are matched through data association strategy to create the corresponding track and update the tracking results. On the other hand, the JDE-based method first extracts the possible targets’ classes, locations and appearance features jointly in a network, and second matches the detection results and tracking results by data association and updates the tracking. Therefore, the SDE-based method can train the most appropriate model for each sub-task. In addition, the tracking module first cuts out the detected object boundary box, and then extracts features to help deal with the scale change of the object. On the contrary, although the JDE-based method can implement three different kinds of tasks such as classification, localization and feature extraction simultaneously using only a single network, the anchor frame in this method is relatively rough, which can very easily generate false detection, especially for small targets or insignificant target features. Although the speed of such methods is relatively improved, the tracking accuracy and the tracking stability is not significantly better than those of the former method, considering the images from the mobile perspective of petrochemical enterprises have characteristics such as wide field of vision, cluttered background, small size of objects occupying the whole image and tiny features, which cause difficulties in target feature extraction and model establishment. Therefore, the SDE-based framework is selected in this study.

In the scenario of petrochemical equipment tracking task, more comprehensive target information can be collected because of the mobile imaging equipment with flexible movement, the wide observation perspective, and the large target search range. At the same time, there are more interference objects, which lead to poor discrimination between targets and background, mutual occlusion among equipment targets. In addition, mobile imaging is restricted by the ground height, which leads to many small targets in the images. Due to its own motion, camera jitter frequently appears in mobile imaging, as well as angle of view transformation and other phenomena, making the tracking target scale change greatly. These problems affect the tracking accuracy and precision in the mobile visual angle.

To solve this problem, a common approach is to integrate common object detectors and trackers to form robust tracking. The integration module usually improves the performance of the model by obtaining target characteristics through a large amount of data training. For example, pedestrian targets in multiple object tracking (MOT) challenge [7] usually obtain large and accurate pedestrian position information through integrated detectors, or improve the tracking effect by reducing the detection threshold and increasing data correlation [4]. However, considering the characteristics of actual targets in different scenarios, as well as the computational complexity and configuration details of deep learning-based multiple object tracking, directly applying a general tracker is not a simple process, nor is it an optimal solution to a specific task. On the other hand, due to the strong geometric structure characteristics of the petrochemical equipment itself, the significant characteristics of the equipment to be tracked can be quickly extracted by designing shallow features, which could increase the convenience of data association. Therefore, improvement of the algorithm’s adaptive ability to the complex petrochemical enterprise working scenarios has become a challenging problem in the research of the SDE-based tracking algorithms.

Based on the above analysis, this paper focuses on improving the two-stage tracking model by designing more specific equipment detectors and stronger data association methods. It builds a new MOT algorithm that jointly optimizes the equipment detector and data association under the mobile perspective in petrochemical working scenes. Specifically, the main characteristic and contributions of this work are as follows:

1. This paper improves the latest YOLOv7 detector [8] by adding attention mechanism and small target detection layer to focus on representing petrochemical equipment under different sizes, and uses feature fusion to carry out multi-scale weighted fusion of features of different scales to solve the problem of large target scale change. It aims to alleviate the detection difficulties of large target scale change and poor small target capturing performance of petrochemical equipment from the mobile perspective.
2. This paper proposes hybrid matching to build data association, which adopts an improved ResNet50 network [9] as the backbone to learn appearance feature representation for improving the network’s ability to perceive tiny equipment, and adds channel and spatial attention modules to effectively extract target key features and strengthen the ability to distinguish differences within classification. It can solve the problem of lost tracking target caused by complex background disturbance and occlusion.
3. This paper also proposes a hybrid similarity measure that incorporates the traditional non-parametric local texture feature descriptor (LBPH) [10], deep metric learning feature, and location feature to carry out data association for equipment pairs, and a reasonable threshold is set to exclude significantly dissimilar key equipment pairs. The experiment on the self-built petrochemical equipment tracking data set verifies the effectiveness of the proposed algorithm, and its tracking accuracy and precision achieve 86.4% and 78. 8%. It also reduces the identity switching times of petrochemical equipment to a certain extent, and could basically satisfy the tracking stability requirements of multiple key equipment from the mobile perspective.

The rest of the paper is organized as follows: Section 2 introduces the related work about the tracking-by-detection algorithms. Section 3 describes our proposed model in detail. Section 4 and Section 5 introduces the data sets and experimental results and proposed discussions. Section 6 is a summary of the paper.

## 2. Related Work

Multiple object tracking is a vital technique for realizing the identification, localization, and tracking of different objects in visual videos, so it has received much attention in the last two decades. With the rapid development of the deep neural network, the performance of multiple object tracking methods has been gradually improved. In particular, the detection-based multi-target tracking method has always occupied the dominant position in recent years, both in academic and industrial applications.

The tracking-by-detection method usually divides the multi-target tracking task into two separate sub-tasks, namely detection and data association. The first step is to obtain the target box prediction of each target through a detector. The second step is to construct the discriminant features for matching through the object appearance, motion state and other information, and associate the objects across frames to form a continuous track. Before the rise of deep learning, the performance of the detector was not powerful enough to locate most targets in the scene; this kind of method mainly focused on the data association. Researchers focused on studying complex data association methods to make up for the shortcomings of the detector. The method in this stage usually models target tracking as a probability assumption maximization problem and representative work includes multiple hypothesis tracking (MHT), particle filter algorithm, Markov decision tracking [11,12], etc. With the gradual improvement of convolutional neural network (CNN), Kim et al. [1] proposed a novel multihypothesis tracking algorithm that models each target using CNN and then optimally matches them with the hypothesis trajectory. Although the performance of this algorithm is improved compared with the original version, the speed is still not high. The first method to rely on high-performance detection results is IOU Tracker proposed by Erik et al. [13], whose basic principle is to measure the intersection over union (IOU) of the previous frame detection frame and the current one, and determine those with high IOU as the same target so as to achieve fast matching.

In the last five years, along with the emerging excellent detectors such as R-CNN series [14], Yolo series [15], CenterNet [16] and other detectors, the detection capability of single-frame image has greatly improved, which also produced some simple and efficient tracking methods that rely on widely investigated detection results. In this context, Bewley et al. [2] proposed a simple online and real-time tracking method (SORT), which combines the two-stage Faster R-CNN detector [17] and simplified tracking strategy and receives much attention. Specifically, SORT predicts the candidate frame of the existing target in the current frame through Kalman filtering, and uses the intersection and merger ratio of the predicted candidate frame and the detection frame to build the matching relationship. This method effectively alleviates the problem of matching failure due to the low intersection and union ratio of the same target caused by the target movement. This algorithm runs faster because of its simple framework. However, its disadvantages are also obvious. In the face of challenges such as frequent occlusion of targets, missed detection and false detection of detectors, and high-speed moving of targets, this algorithm is prone to cause problems such as error matching or track fracture. At the same time, when the SORT algorithm handles exchanges and occlusions among targets, there is a defect of target ID switch or generating a new track, that is, the continuous tracking for tracked targets is lost, so it cannot perform stable tracking for a long time.

Aiming at solving the problem of unstable target identity of SORT algorithm, Wojke et al. proposed a DeepSORT [3] algorithm that improves SORT by taking the object’s appearance features into consideration. It introduces an additional re-identification (ReID) network to model the appearance of each target in the scene and adds a cascade matching strategy. Due to the introduction of appearance information, DeepSORT is more robust in missed detection or fast-moving target scenarios, and its tracking performance is significantly improved in many scenarios. However, the ReID module of this method does not reflect the difference among different targets when modeling the appearance of each detection frame separately. Because of the uncertain number of targets in the real videos, the time for extracting target features is not fixed, which easily leads to unstable frame rate. In the context of object ReID, Bayraktar et al. proposed an embedding generation module which is tightly coupled with the object detection module and a triplet-based matching module to achieve the fast Re-ID task of objects [18]. A merit of this model is the rich object encoding in the instance segmentation which can be directly shared to the module for training a more discriminative representation via a triplet network.

In the two modules of detection and data association, in order to effectively reduce the task competition between detection and ReID, Liang et al. proposed a cross-association network [19] to learn independent task expression and increase the collaboration between tasks. The method first decouples detection and ReID tasks into two independent branches to learn independent task representation, then uses self-attention mechanism to make self-correlation and cross-correlation between the features of the two tasks. The self-correlation promotes independent task learning, while the cross-correlation promotes collaborative learning. At the same time, in order to solve the problem of target scaling, a scale awareness network is introduced to enhance the influence of target-related embedding under different resolutions. Finally, different high-resolution features are integrated as output to help learning scaling awareness expression. More recently, Suljagic et al. proposed an interesting similarity-based person ReID framework by adopting a Siamese neural network via shared weights [20]. Once detections are performed and the Siamese feature extraction is obtained, a similarity array for assessing tracks and detections is computed to obtain the ReID results. Wang et al. proposed a group-guided data association for multiple object tracking [21], where the data association is divided into intra-group and inter-group. For the intra-group, detections are recovered and associated by min-cost network flow. Meanwhile, For inter-group, a hypotheses is associated to solve long-term occlusion and reduce false positives.

The latest ByteTrack [4] proposes a simple and efficient data association method to handle the problem of insufficient data association in SORT algorithm. This algorithm removes the background from the low-score detection results while retaining the high-score detection results by using the similarity between the detection frame and the trackers. It can discover the difficult real objects (such as occlusion and blurring) using the low similarity so as to reduce missed detection and improve the continuity of the track process. More recently, a hybrid motion model for multiple object tracking has been proposed to measure optical flow similarity and transition smoothness [22], where the tracker motion is described by the multimode motion filter for adaptive modeling and the data association is implemented by a spatiotemporal evaluation mechanism, making it achieve high discriminability in motion measurement.

As seen above, multiple object tracking in complex scenes is a very challenging task. To accurately track the historical trajectory of the targets, not only appropriate measurement elements but also appropriate tracking logic are required. The Bayesian nonparametric modeling is also introduced to predict dynamic dependencies in MOT [23]. Due to the diversity of scenarios in real petrochemical working places, the design of MOT algorithm needs to consider various exceptions to ensure the accuracy of tracking results to meet the needs of such scenarios. The current mainstream algorithms rely on target detection, and need to extract the representation vector from the image data in the target frame. 

## 3. Proposed Method

In this section, we describe the details of the proposed petrochemical equipment tracking model using an improved Yolov7 network and hybrid matching. We first present the main structure of our model that combines the detection and tracking stages to handle the sequential video frames, and then explain the major submodules, such as the improved Yolov7 network, appearance modeling by Siamese network with attention mechanism, and optimized data association and cascaded matching.

### 3.1. Main Structure

**Figure 1:**
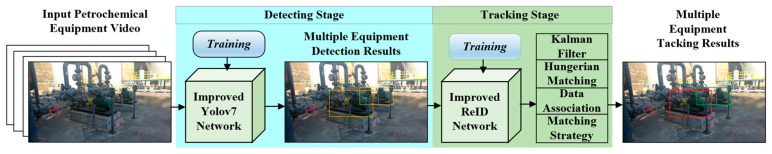
Flowchart in our model.

Figure 1 depicts the overall flowchart of the proposed model. It consists of detection and tracking modules and it works as follows. For a video that contains petrochemical equipment and needs to be tracked in the onsite inspection and maintenance working scenes, its first frame is sent to the equipment detection module, and the improved Yolov7 detector detects the pieces of equipment, assigns them identities and records their locations, as well as some deep features extracted by the ReID network and traditional features. Then, for a second frame, the Yolov7 detector still detects equipment and produces the same information. These kinds of information are further sent to the tracking module, where a hybrid matching is built to associate different equipment among the previous records and current results. It is noted that the movement information of each equipment is estimated by Kalman filter. Then, the equipment assignment is obtained via Hungarian algorithm and the different tracks are built. In the following sections, we present the details of our framework.

### 3.2. Improved Yolov7 Network

The “tracking-by-detection” strategy for multiple object tracking first sends the input image to the detector for identifying and localization targets and then tracks them according to the detected target. The tracking performance largely depends on the detector effect, so a powerful target detector is particularly essential in the whole tracking process. The flowchart of traditional object detection algorithms is as follows: Firstly, the sliding window is used to traverse the image in the input image to obtain the possible target areas and candidate boxes. Secondly, the image features in the candidate box are extracted and converted into feature vectors. Common traditional feature extraction algorithms include gradient histogram, local binary descriptor, etc. Finally, the target object and corresponding category are determined according to the feature vector. Traditional target detection algorithms generate a large number of redundant windows when candidate boxes are selected, and then generate redundant calculations, which deteriorate the speed and performance of the entire algorithm. In addition, traditional algorithms can only extract low-level features, and ultimately cannot obtain the global optimal solution.

In recent years, the object detection algorithm based on deep learning has developed rapidly. Compared with traditional algorithms, it not only improves the overall detection speed, but also can identify the target with higher accuracy and therefore becomes the mainstream of the current object detection field. The deep learning-based object detection algorithms can be divided into two-stage- and one-stage-based types. Two-stage detection algorithm contains region proposal in the detection process where the images in the candidate box generated by region proposal are classified and located by convolutional neural network. The representative algorithms are Faster R-CNN [24], Cascade R-CNN [25], etc. On the other hand, the one-stage detection algorithms use regression method to infer the bounding box and related category probability. Representative methods include you only look once (Yolo) series [15,26,27,28,29], single shot detector (SSD) [30], RetinaNet [31], EfficientDet [32], etc. By using a single end-to-end network to complete the output of target positions and categories, the detection speed of the entire algorithm has been greatly improved. At the same time, because there is no more accurate candidate area, the detection accuracy of the one-stage algorithm is correspondingly reduced. However, with the improvement of computer performance and the continuous development of in-deep learning, the network structure of the one-stage detection algorithm has been continuously optimized and its performance has been gradually improved. Among the algorithms, Yolo [15,26,27,28,29] has become mainstream, and the recent version Yolov7 [8] has both fast detection speed and high accuracy, competitive with Faster R-CNN. Therefore, in our model, Yolov7 is chosen as the basic detector framework and improved according to the characteristics of petrochemical equipment. Specifically, the structure of the network in our model is as follows.

#### 3.2.1. Network Structure

**Figure 2:**
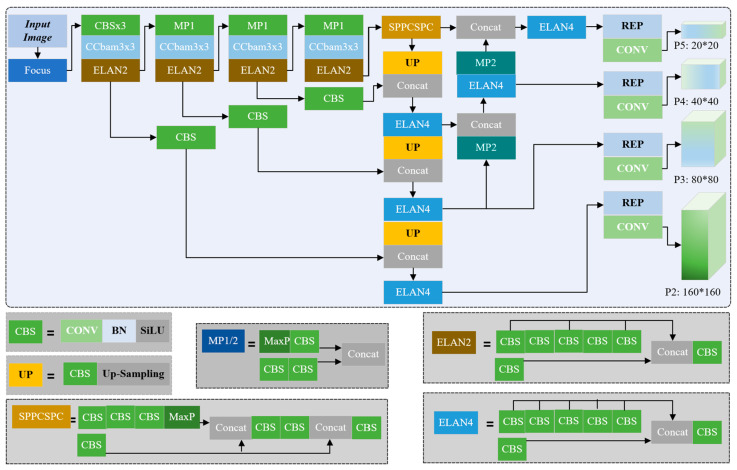
Improved Yolov7 neural network structure in our framework.

Figure 2 illustrates the structure of our improved Yolov7. It can be divided into three parts. In the backbone, there are several CBS blocks that denote convolution, batch normalization, and SiLU operation, Cbam block that builds the channel and spatial attention, and ELAN2 or ELAN4 block that integrates CBS in different ways such as the residual block. There are four branches from the backbone to the neck part for feature fusion, just like the feature pyramid networks (FPN) [33] and path aggregation network (PANet) [34] for feature map with four different scales. In this way, the network can learn the features corresponding to the equipment with different sizes. Finally, these features are sent to the third part to build the detection head. Considering the complicated background and severe occlusion in the scenario of petrochemical working place, an attention mechanism named Cbam is added to the backbone to extract the ambient features.

#### 3.2.2. Cbam Attention Module

In the field of computer vision, attention mechanism is widely used in image classification, object detection, segmentation tasks, etc. Essentially, the attention mechanism is to determine the candidate areas that need to be focused on, and enhance the information of the focus area in the image through a series of weight parameters to extract more details of the target while suppressing some useless information. The SENet [35] is a famous channel mechanism that optimizes specific category of feature information by modeling the correlation of image feature channel domain, but SENet only considers the coding of information between channels, ignoring the importance of spatial information. Then, the ECANet [36] is proposed to improve the SENet by adding a local cross-channel interaction strategy without dimension reduction and adaptively selecting the size of one-dimensional convolution kernel, thus achieving performance optimization. Cbam [37] is a combined attention mechanism that models attention separately from channel and space. It includes a channel attention module that follows the SENet with an extra max-pooling layer and a spatial attention module that are characterized as a lightweight structure. In this way, it can save parameters and computing power, and ensure that it can be integrated into the existing network architecture as a plug-and-play module. Previous Yolov5 was motivated by the attention mechanism in this way [38]; in practice, the mechanisms can be combined to be added to the existing network in a parallel or serial order.

**Figure 3:**
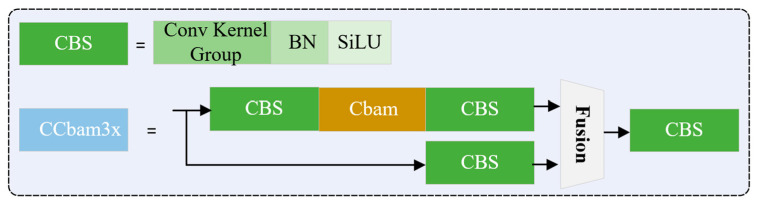
Cbam attention module in our detection network.

Figure 3 shows the structure of Cbam that is incorporated into our improved Yolov7 network. The order is as follows: serial channel first, and then the use of space information. This module is added to the convolutional layer for making the equipment heavier intentionally and helpful for correcting misdetection.

### 3.3. Appearance Modeling by Siamese Network with Attention Mechanism

Appearance modeling refers to using the appearance information of the target to extract the distinguishing features through some operator or deep convolution neural network so as to ensure that a multi-object tracking algorithm can match each target stably and prevent target switching or loss. The current mainstream methods all adopt advanced re-identification technology (ReID) to achieve appearance modeling, that is, use well-designed neural networks to abstract each object into a feature with higher-order discriminant semantics. In the scene where object occlusion occurs frequently, it can effectively correct the mismatching by the spatiotemporal matching based on IoU [13]. At present, appearance modeling has become the core technology in multi-object tracking, and also one of the most effective ways to improve the ability of model data association.

The ReID technology is not improved in SORT algorithm. DeepSORT adds the object appearance features to the associated cost calculation by using a simple CNN to extract the appearance features of objects in the detected frame, and calculates the similarity through cosine similarity. However, this method does not take the differences across objects into account. Furthermore, the apparent feature extraction in DeepSORT uses the ReID network structure in the field of pedestrian re-recognition. The network lacks discrimination in feature extraction, and multiple pedestrian tracking always shows different sizes but without extreme variety. Therefore, matching errors are likely to occur during state transition, which affects the tracking accuracy.

**Figure 4:**
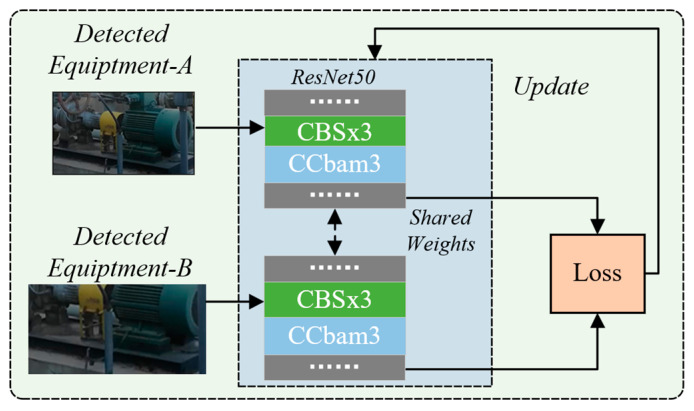
Improved Siamese ReID neural network structure in our framework.

To handle this problem, the Siamese network is introduced to learn the discriminant deep feature of petrochemical equipment. Traditional methods of target tracking based on the Siamese network treat the image features of each layer and channel equally. However, the semantic information contained in the shallow and deep features is different, and the contribution of the information contained in each channel is also different. Different from them and considering the difficulty of extracting features under occlusions, the ResNet50 network is chosen as the backbone in our ReID network and its flowchart is depicted as Figure 4, where the Cbam module is also incorporated into the middle of CBS block. Therefore, in this network, different weights are assigned to features according to their importance, which makes the network pay more attention to features that are conducive to target tracking and ignore useless information. The working principle of the attention weight factor group generation module is to receive the multi-layer response maps calculated by the two branches, respectively, generate the corresponding attention weight factor group according to the richness of the target information contained in it, act on the response map according to the channel and conduct feature fusion to obtain the final similarity result.

### 3.4. Optimized Data Association and Cascaded Matching

The main task of the tracking module is to design proper correlation measure and matching method. At present, the correlation measure method is mainly established by using appearance similarity, motion state information, location relationship, etc. The association methods are mainly divided into two categories: offline and online association. Offline association uses the target information in all-time series to globally optimize the association of targets and tracks, while online association only uses the current frame and history frame information for association. In the mobile camera viewpoints, the multi-object tracking algorithm can only capture images frame by frame, so the online correlation method is more suitable for multiple equipment tracking in the mobile camera perspective. DeepSORT uses more reliable correlation measurement and data association strategy on the basis of SORT, which can effectively track for a long time and reduce identity switches in the tracking process to a large extent.

Specifically, DeepSORT combines the target motion state information and appearance features for correlation measurement to achieve a relatively stable multi-target tracking state. The Kalman filter plays an important role in DeepSORT for predicting the state of next frame because it has both fast speed and high accuracy. However, there are a lot of motion uncertainties in the moving camera viewpoint, such as camera jitter caused by the impact of road bumps on the camera platform and the running speed of the mobile camera platform itself. As a result, the Mahalanobis distance between two adjacent frames of the same device is still large under the moving perspective. Even if the matching is correct, it may be misjudged as different equipment, which ultimately causes matching failure. Therefore, the association measurement should focus on the appearance feature information. At this time, the appearance modeling in the tracking module largely determines the final tracking result.

With the rapid development of computer vision, the feature extraction network based on deep learning shows its superiority and particularity. Compared with traditional methods, it can learn and extract the features of objects more completely and reliably. DeepSORT uses an 11-layer CNN to output 128-dimensional target feature vectors. This network can only extract more obvious features. For some subtle features, the ability to extract them is poor. However, the target scale changes greatly in the mobile camera perspective, and the differences between targets may not be obvious in the image. Therefore, this paper proposes a hybrid matching strategy for data association between targets. Specifically, the similarity functions constructed in this paper include three categories.

(1) LBPH-based similarity

Local binary patterns histograms (LBPH) is a powerful feature descriptor that arises from the basic local binary patterns (LBP). It can encode an image into a simple and computationally efficient nonparametric local texture feature descriptor. Because of its high feature discrimination and low computational complexity, it is famous for being able to recognize faces from the front and side. It has advantages of not being affected by lighting, scaling, rotation and translation, which makes it very suitable for describing the equipment in a fast and discriminant way.

The basic process of establishing LBPH feature of petrochemical equipment is as follows: first, each pixel is taken as the center, the relationship with the gray value of surrounding pixels is judged, binary coding is carried out, and the LBP encoded image of the whole equipment image is obtained; then, the LBP image is divided into regions, the LBP coding histogram of each region is obtained, and the LBP coding histogram of the target is further obtained. Finally, the similarity is calculated by comparing the LBP coding histograms of different targets, that is,

(1) $$d_{1}\left(i,j\right)=\sum_{k=0}^{len}\sqrt{h_{i}^{\left(k\right)}q_{j}^{\left(k\right)}},$$

where $h_{i},q_{i}$ are the coefficients of the *k*th bin in the histogram of the *i*th object and *j*th object, respectively. Len is the maximum value of the histogram. $d_{1}\left(i,j\right)$ denotes the Bhattacharya coefficient and its range is between 0 and 1.

(2) Position similarity

In this paper, Mahalanobis distance that modifies that correlation and scale variation in data resided in high dimensional space is used to perform similarity motion matching between the equipment detection position and the predicted equipment state:

(2) $$d_{2}\left(i,j\right)={\left(d_{j}-y_{i}\right)}^{T}S_{i}^{-1}\left(d_{j}-y_{i}\right),$$

where $y_{i}$ denotes the position of the *i*th predicted equipment and $d_{j}$ is the bounding box of the *j*th object. $S_{i}^{-1}$ is the inverse covariance matrix obtained by Kalman filter in current frame.

(3) Deep appearance similarity

In order to prevent the lost target from re-entering the field of vision and the occurrence of identity conversion, the deep appearance feature within a number of frames is reserved and denoted as $R_{i}$. The number of elements is set as 100 in this paper. For the measurement of the appearance characteristics of the detection results and tracks, our algorithm adopts the minimum cosine distance to measure the feature distance between the two features:

(3) $$d_{3}\left(i,j\right)=\min\{1-r_{j}^{T}r_{k}^{i}|r_{k}^{i}\in R_{i}\}\;,$$

where $d_{3}\left(i,j\right)$ is the minimum distance between the feature of *j*th detected equipment and all the features of the *i*th trackers in the tracking set $R_{i}$. In order to improve the feature extraction ability of the original network, the ResNet50 network is introduced to output 2048-dimensional target feature vectors to enhance the network’s ability to extract petrochemical equipment’s deep features. In addition, the spatio-channel attention mechanism is added to the network to improve the network’s ability to re-identify and reduce the tracking loss problem caused by long-term occlusion of key equipment under the mobile perspective, and the cross-entropy loss function is implemented to enhance the ability to measure the similarity of equipment.

(4) Hybrid matching measurement

The hybrid matching measurement is built as follows:

(4) $$D_{i,j}=\beta_{1}d_{1}\left(i,j\right)+\beta_{2}d_{2}\left(i,j\right)+\left(1-\beta_{1}-\beta_{2}\right)d_{3}\left(i,j\right),$$

where $\beta_{1},\beta_{2}$ are the weights of different kinds of similarity, and they are settled as $\beta_{1}=0.2$, $\beta_{2}=0.2$ according to experiments. $D_{i,j}$ is the hybrid measurement, and a lesser value means a larger similarity for the *i*th track and the *j*th detection results. To reduce mismatching, we further divide the similarity into two parts and build the data association value as follows:

(5) $$c_{i,j}=\delta \left(d_{1}\left(i,j\right),t_{1}\right)\delta \left(d_{2}\left(i,j\right),t_{2}\right)\delta \left(d_{3}\left(i,j\right),t_{3}\right)\;D_{i,j},$$

where $\delta \left(d,t\right)$ is an indicator. It is 1 when d<t and 0 otherwise. The $\chi^{2}$ distribution is borrowed to set these thresholds to judge the similarity between the ith track and the jth detection result and *t* is chosen as the 95% confidence in $\chi^{2}$ distribution. In our experiments, $t_{1}=0.937$; $t_{2}=0.841$; $t_{3}=0.906$. By this way, the tracking accuracy and precision of the algorithm could be improved, and the identity switching frequency can be reduced efficiently.

**Figure 5:**
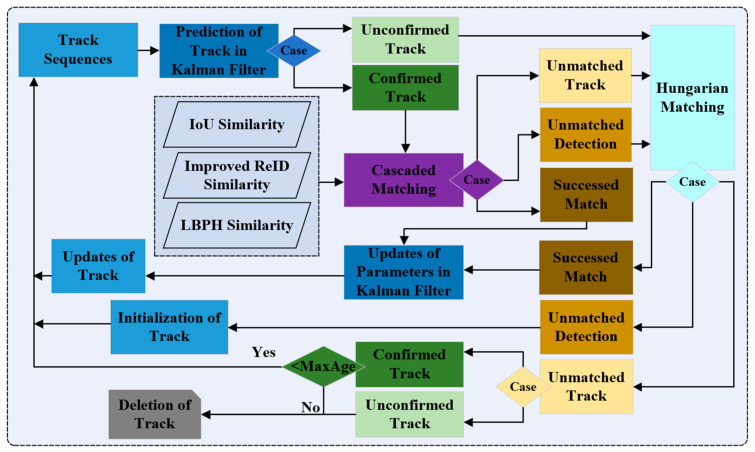
Cascaded matching flowchart in our framework.

Based on the above calculation results, the cascaded matching strategy is further incorporated to build multi-equipment trajectory, Hungarian matching algorithm is implemented to perform correlation matching for multiple equipment according to the hybrid matching results [39], and the optimal tracking trajectory is finally obtained as this frame. The detail of the tracking flowchart is depicted in Figure 5.

## 4. Experiments

In this section, we provide the implementation and experimental details of the proposed model. Specifically, we first introduce a new self-built video data set of petrochemical equipment and the construction of data set for multiple petrochemical equipment tracking, then we offer the evaluation metrics for detection and tracking tasks, and finally we report our experimental platform and training details.

### 4.1. Petrochemical Equipment Data Set

In our research, a new video data set of petrochemical on-site inspection and maintenance scene was collected and labeled. The data set contains video sequences of five different petrochemical enterprises, including five key petrochemical pieces of equipment: screw pump, centrifugal pump, heat exchanger, spherical tank, and vertical oil storage tank. The videos were captured from the moving angles of view includes such shooting difficulties as angle transformation, camera shake, camera motion, illumination change, background interference, etc. In our experiments, one frame image is extracted every five frames to form a video sequence from the video with a frame rate of 30 frames per second. In practical application, the multiple petrochemical equipment tracking framework needs to infer the current frame image, and then extract the image at the current time of the video. The processing speed of the constructed tracking algorithm is 10 to 15 frames per second, which is about 1/2 to 1/3 of the video frame rate. Therefore, the constructed video sequence conforms to the actual application scenario of multi-target tracking algorithm in on-site inspection and maintenance of petrochemical enterprises. Table 1 reports the detailed data set information.

### 4.2. Evaluation Metrics

To evaluate the performance of the proposed algorithm, the AP (Average Precision) and *mAP* (mean Average Precision) are used for multiple object detection experiments and CLEAR MOT for multiple object tracking, where *AP* denotes the area that covers the Precision–Recall (P-R) curves and $Precision=\frac{TP}{TP+FP},\;Recall=\frac{TP}{TP+FN}$. In the formula, *TP* is the numbers of objects being correctly detected and FN is the numbers of missing objects; *FP* is the number of objects being falsely detected; *mAP* is the average of the *AP* values of all subjects. That is $\;mAP=\frac{1}{N}\sum_{k=1}^{N}AP_{k}$.

The CLEAR MOT evaluation metric [40] mainly includes the following terms. ML (Mostly Lost Tracked) is defined as the ratio of ground truth trajectories that are covered by any track hypothesis for at most 20% of their respective life span. MT (Mostly Tracked) is defined as the ratio of ground truth trajectories that are covered by any track hypothesis for at least 80% of their respective life span. IDSw (IDentity Switches) is defined as the number of times a track switches from one ground truth item to another. A lower IDSw means a better performance of consistent tracking. FPS (Frames Per Second) is defined as a quantitative measure of how tracking result is displayed in a motion video. A higher FPS results indicates a smoother and more fluid visual tracking experience.

*MOTP* (Multiple Object Tracking Precision) is defined as

(6) $$MOTP=\frac{\sum_{t,k}d_{t}^{k}}{\sum_{t}c_{t}},$$

where $d_{t}^{k}$ denotes the distance between detected objects$\;O_{k}$ in the *t*th frame and the predicted objects, $\;c_{t}$ is the successful number of matched objects in the *t*th frame. *MOTP* is closely related to the accuracy of object detection, and it reflects the localization accuracy of object detection. The accuracy is higher when the value is close to 1.

*MOTA* (Multiple Object Tracking Accuracy) is defined as

(7) $$MOTA=\frac{\sum_{t}\left(m_{t}+fp_{t}+mme_{t}\right)}{\sum_{t}g_{t}},$$

where $m_{t}$ denotes the number of objects being missed; $fp_{t}$ (false positive) is the number of false detections at the *t*th frame. $mme_{t}$ (mismatches) is the number of mismatched objects at the *t*th frame. *MOTA* is independent from the accuracy of object detection, and it aims to evaluate the tracking algorithm’s performance on keeping the tracks. The accuracy is higher when the value is close to 1.

### 4.3. Experimental Platform and Training Details

All experiments in this paper were conducted on the Ubuntu18.04LTS system, which has 2.0 GHz Intel CPU and 48 GB RAM. The GPU is NVIDIA RTX 2080Ti. The program environment is Anaconda 5.0.1 (Python 3.7) and PyTorch1.7. In this paper, the improved network structure of Yolov7, and Siamese network with ResNet50 backbone are used for training. The Adam optimizer was chosen. The initial learning rate was set at 0.001, the batch size set at 32, and the number of learning epochs was set at 150. For detection, the input image was resized as 640*630, the confidence for detection was 0.3 for enough detection bounding boxes. For tracking, the initial frame number was 3, and maximum number of keeping the lost frames was 30, and the number of frames for computing the appearance features was 100.

## 5. Results and Discussion

In this section, we present the experimental results of proposed model and related algorithms in different respects and then offer some discussions. Specifically, we first present the experimental results of multiple equipment detection by the detection module of proposed model and related detect algorithms. Then, we report the experimental results of our ablation study. Furthermore, we provide the comparatively experimental results of proposed model and related tracking methods. Based on these results, we offer some discussion from several viewpoints.

### 5.1. Experimental Results of Multiple Equipment Detection

This experiment first verified the performance of the improved Yolov7 equipment detector and selected various petrochemical equipment in the petrochemical equipment training data set to form object detection data set for offline training of the detector. Since there are five categories of equipment tracked on the petrochemical video data set, the average accuracy of these five categories of detection is concerned with the object detection network, and *mAP*50, *mAP*75 of all categories. They denote the mean *AP*s under different IoU (0.5 and 0.75).

In order to evaluate the characteristics of the improved Yolov7, some state-of-the-art two-stage and one-stage detection algorithms were chosen in the experiments, including Faster RCNN, SSD, RetinaNet, EfficientDet, Yolov5x and Yolov7. The parameters and experimental setting followed the original model and the pretrained model on COCO data set [41] were also employed. We follow the method [38] for details of some improved versions of these algorithms.

Table 2 reports the comparative results of these algorithms, where “*” indicates there are difference in mean between the proposed model and the compared one in *t*-test at 95% significance level. From there, it can be observed that the algorithms in Yolo series perform better than the two-stage Faster RCNN and one-stage SSD (single-shot detector), also the latest RetinaNet and EfficientDet. Yolo5x has achieved high detection accuracy at 97.4% and 95.6% in *mAP*50 and *mAP*75, with a leading 1.2% and 0.8% in comparison to EfficientDet, which means the reasonability of the proposed algorithm in model selection. In addition, the detection accuracy of proposed detection model is significantly better than those of Faster RCNN, SSD, RetinaNet, EfficientDet and Yolo5x in terms of mAP50, and it outperforms them all in terms of mAP75 and the Centrifugal pump.

As for the detection algorithms in Yolo series, Yolov7 outperforms Yolov5x in mAP50 and mAP75 for 0.2% and 0.5%; nevertheless, a decrease of 0.1% accuracy in detecting spherical tanks. The performance of our improved Yolov7 is better than that of Yolov7 and Yolov5x, and it increases by 0.1% and 0.4% in *mAP*50 and *mAP*75 compared with Yolov7. The detection of each type of equipment was improved or maintained, and the detection accuracy of spherical tank improved the most, reaching 0.3%. It has 0.3% higher *mAP*50 and *mAP*75 in comparison to Yolov5x, and the most improvement occurrs in detection for centrifugal pump, reaching 0.4%. As for the tracking task in our research, a detector with high accuracy is a perquisite, so this experiment verifies the effectiveness of the improved Yolov7 and the reasonability of incorporation of spatial and channel attention mechanism for focusing on the petrochemical equipment.

### 5.2. Ablation Experimental Results

Our proposed algorithm mainly includes three innovative modules, namely Yolov7-CA for target detection, SiameseNet-CA for appearance feature learning in ReID, and hybrid similarity calculation for data association. In order to verify the impact of these three modules on the overall performance of the algorithm, we constructed three variants by deleting specific modules: V1—This variant only uses Yolov7-CA, and the corresponding module of DeepSORT algorithm is used in the ReID and data association modules; V2—This variant only uses SiameseNet-CA, and the other modules use the corresponding modules in the DeepSORT algorithm; V3—This variant only uses the mixed similarity calculation, and the other modules use the corresponding modules in the DeepSORT algorithm. On this basis, we carried out ablation experiments, and the results are shown in Table 3.

It can be seen from Table 3 that after the detector is improved, it makes the largest contribution to the improvement of tracking performance, which is 0.4% higher than that of DeepSORT in terms of *MOTP* and *MOTA* metrics, indicating that an accurate detector is of great value for improving tracking accuracy. Then, after using SiameseNet-CA in ReID, these two metrics increased by 0.3%, which can effectively extract appearance templates that are consistent with the appearance of the current frame target and eliminate low-quality templates. This is an independent benefit after improving the appearance similarity identification ability. After using the hybrid similarity measurement, the performance is improved by 0.2% and 0.3%, but this approach has the least impact on the reduction in tracking speed. These three modules are integrated to form the proposed algorithm, which has achieved greater improvement in the overall performance.

### 5.3. Experimental Results of Multiple Equipment Tracking

The tracking experiment was conducted with comparison to five related state-of-the-art multiple object tracking algorithms, including SORT, DeepSORT, ByteTrack, FairMOT and CenterTracker. SORT was proposed by Bewley et al. [2], and it is a baseline in “tracking-by-detection” category. DeepSORT was proposed by Wojke, and it employs CNN for feature extraction and reduces the wrong identity switches. In our experiments, it is taken as the baseline. ByteTrack is the latest SDE-based tracking algorithm that focuses on the simple and efficient data association by adjusting the thresholds for detection and improving the consistency of tracking. FairMOT is the latest JDE-based tracking algorithm that performs object detection based on anchor-free detection method. CenterTracker is also a very recent JDE-based tracking algorithm that employs the points instead of bounding box to represent the objects.

Table 4 reports the experimental results on our petrochemical equipment data set. It is seen that the proposed algorithm achieves the most competitive performance in the evaluation metrics. Specifically, it obtains 86.4 in *MOTP* that represents the overlapping degree between the detected equipment and the ground truth objects, 0.8% higher than that of DeepSORT. Considering the *MOTP* is related to the ability of detection module in the whole algorithm, it has further verified the advantage of the improved Yolov7 model. The proposed model obtains 78.8% value in terms of *MOTA*, same as ByteTrack and 1% higher than DeepSORT. According to the MT evaluation, the proposed algorithm takes the second place and ByteTrack takes the first, with 3% higher value than DeepSORT. Considering MT is an indicator for long-term tracking, this result shows the improvement of our ReID network in regard to support for data association.

The proposed algorithm also achieves best results on ML and IDSw, and the numbers 33 and 140 are lower than those in DeepSORT (35 and 186) and ByteSORT (34 and 142). A lower ML means less lost tracking, and lower IDSw means more consistent tracking. In the moving viewpoints of the petrochemical working scenes, due to the occlusion of irrelated equipment and camera jitter, the key petrochemical equipment does not always appear stably in the video. The proposed algorithm has the smallest total number of ML and IDSw, which shows that it has a good ability to maintain the benchmark tracking state, as well as the comprehensive performance of target detection and cascaded matching.

In comparison with other algorithms, it can be seen that the performance of basic SDE-based trackers, such as SORT, is not as good as that of trackers such as CenterTrack, and the differences in indicators such as *MOTP* and *MOTA* are 2.5% and 1.6%. However, the improved versions of SDE-based trackers, such as DeepSORT, are slightly better than CenterTrack, and the improvements in *MOTP* and *MOTA* are 0.4% and 0.5%. The latest SDE-based ByteSORT algorithm performs better than the recent representative JDE-based FairMOT. This is mainly because the target change scale of key petrochemical equipment is large from the moving perspective, and it is difficult to obtain the regional information of equipment by using the target center point.

It is worth noting that in terms of measuring the tracking speed of tracking algorithms, SORT algorithm and JDE-based CenterTracker have achieved significant advantages, which are obviously superior to the proposed algorithms and ByteTrack. This is mainly because these algorithms use simple and effective data association strategies. The above results show that the proposed algorithm has achieved competitive results on five data sets, which can effectively reduce the number of missed equipment detection and track disconnection and is conducive to improving the tracking accuracy and stability of the multi-object tracking algorithm.

**Figure 6:**
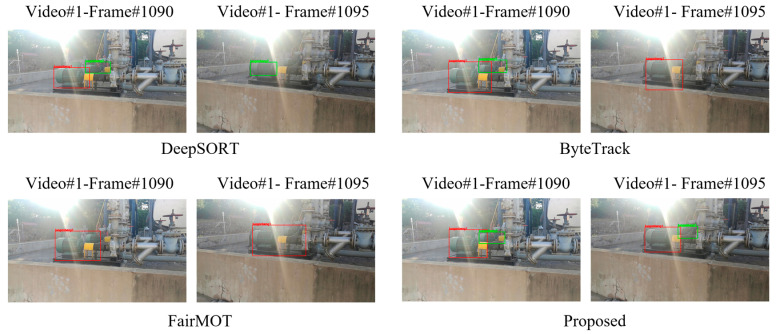
Tracking examples of screw pump by four typical compared algorithms.

We qualitatively examined the performance of our model and other methods. As shown in Figure 6, the tracking effects of different representative algorithms on the video of the screw pump can be visually compared and analyzed. It can be seen that the video presents a petrochemical enterprise on-site environment with uneven lighting under strong light. The screw pumps are located in a complex petrochemical working background, and there is a serious occlusion phenomenon. DeepSORT algorithm can identify the larger screw pumps under strong light, but omits the smaller screw pump with serious occlusion, and the tracking screw pump ID is switched incorrectly. The ByteTrack algorithm can identify the front and rear screw pumps at frame 1090. However, at frame 1095, the smaller screw pumps are lost. The FairMOT algorithm can also identify the front screw pumps at frame 1090, but along with the tracking process, the information of the smaller screw pump object is lost. The proposed algorithm successfully tracks the two screw pumps at the 1090 and 1095 frames, reflecting the difference between the two screw pumps in tracking size, and showing good tracking performance.

**Figure 7:**
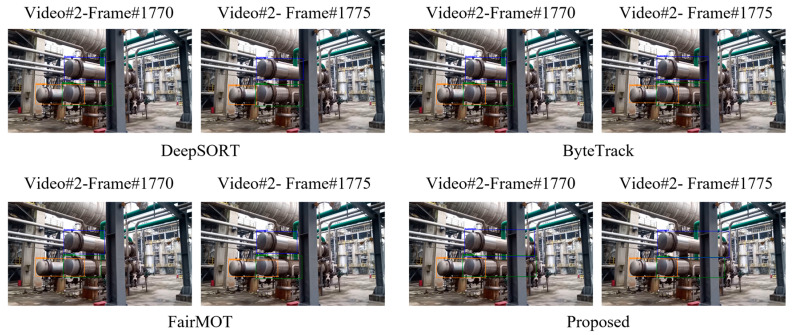
Tracking examples of heat interchanger by four typical compared algorithms.

We further qualitatively examined the tracking effect of our model and other three representative algorithms on the heat exchanger video from another aspect, and the results are illustrated in Figure 7. It can be seen from the figure that the video is derived from the outdoor huge heat exchangers working on-site of the petrochemical enterprises, and complicated background and light introduce many challenges for heat exchanger tracking. In terms of uneven lighting, it is slightly weaker than that of the previous video, but the background is extremely complex, with serious homogeneous equipment and occlusion. The heat exchangers are located in the background of complex petrochemical pipeline environment, and there is a serious shielding phenomenon. DeepSORT, ByteTrack and FairMOT algorithms can identify these three heat exchangers in a complex background, but there are errors in the sizes of the heat exchangers, especially in the length. This is mainly due to the existence of larger columns in the background, which makes the heat exchangers split into two sections, and the original sizes have not been restored in the tracking. Although the proposed algorithm also misidentified the length of the leftmost heat exchanger, it restored the length of the two larger heat exchangers. This is mainly due to the attention mechanism added to the detection module and the appearance feature learning module, and the accurate hybrid matching for data association of devices, which can restore the sizes of the heat exchangers from partial occlusion and improve the tracking performance and tracking retention performance to a certain extent.

### 5.4. Discussion

#### 5.4.1. Overall Discussion

The key equipment tracking task in the field of petrochemical enterprises is among those in the complex industrial working background. The size of the key equipment changes greatly and the occlusion is serious. In order to achieve accurate tracking of multiple key equipment, a separate detection and tracking strategy is chosen in the whole model. By improving the detection model, ReID network and data association hybrid similarity calculation, the accuracy of the equipment detection model and re-identification model is improved, which benefits the overall performance of tracking. Currently, most tracking algorithms focus on the general detection model, ignoring the requirements for detector performance under specific tracking tasks in industrial working scenarios. For this reason, the proposed Yolov7 model combined with channel and spatial attention mechanism is adopted to improve the detection accuracy, which makes the detection accuracy improved by 0.3% to 2% compared with Faster RCNN, Yolov5x, Yolov7 and other models, and leverage the accuracy of various petrochemical equipment to varying degrees. The t-distribution test also verifies the meaningful improvement of the proposed detection model in terms of *mAP*50 and *mAP*75. Thus, it lays a good foundation for the subsequent cascaded matching and Kalman filtering.

Because the traditional recognition model itself has a high demand for features, it directly learns and detects the feature information extracted from visual hints of the images, resulting in poor quality of the generated recognition features. In this paper, however, a Siamese neural network model with channel and spatial attention mechanism is designed to learn the deep features required in target tracking, to distinguish the needs of different instances of the same category, and effectively use the appearance features to improve the accuracy of the tracking algorithm. The experimental results show that this improvement can increase the tracking accuracy by 0.3% compared with the classical algorithm. The neural network in this part can be trained offline and used directly in the actual tracking task, so the impact on the speed of the tracking algorithm is not obvious, and it reduces 0.8 FPS compared with the benchmark algorithm.

In order to speed up the similarity matching between objects, our proposed algorithm uses hybrid similarity to associate the data, which includes the image deep appearance features, the texture features composed of LBPF, and the location features composed of bounding boxes. This is performed by selecting the combination of traditional features and deep ones to let the two models learn the needed feature, and ensure that the recognition model focuses more on learning the low-level appearance features. To sum up, multi-level matching can robustly solve the problems of occlusion, loss, task object deformation among multiple objects to some extent, especially the problem of increasing the number of equipment switching caused by the above problems. This makes our proposed algorithm have a good effect in dealing with a situation in which the equipment is occluded, the trajectory is maintained, and the fragmented trajectory is handled in the petrochemical enterprise working scene.

The feasibility and rationality of our proposed algorithm were also verified through comparative experiments. The experimental results show that the *MOTP* of our algorithm reaches 86.4%, *MOTA* reaches 78.8%, the number of IDs decreases to 160, and the speed reaches 25.2 frames per second. Compared with the other five advanced tracking algorithms in the experiment, the *MOTP* index is slightly lower than the algorithm using SORT without using the target features, because this index only calculates the precision of the tracking box between the target bounding box on the track and the bounding box in the real data set. Therefore, the proposed multi-object tracking algorithm based on optimized detector and hybrid similarity multi-level matching can effectively improve the accuracy of multiple petrochemical equipment tracking and reduces the number of identities switching.

In our practice, the system to deploy this approach is based on the server and mobile device separated network architecture. The videos are captured from a mobile device with a parameter of 24 frames per second (FPS) and a maximum resolution of 2800 × 1500 pixels. Considering that the mobile device with limited processing and storage capacity cannot satisfy the computing requirements, the images are sent to the data server, and then sent to our model in the computing server for processing in a same wireless local area network (WLAN). The tracking speed of our model achieves 25.2 FPS, slightly higher than the basic need of 24 FPS, which can roughly meet the requirements of real-time tracking visualization in the server. It is noted that the data server and the computing server are the same in the whole system. Therefore, the tracking results of multiple petrochemical equipment can be clearly visualized and fluently observed, even if there is some time delay in the image transmission in the WLAN and the image reading, which is usually about 10.5 and 1.6 milliseconds in the testing under a gigabit Ethernet (GigE) industrial standard.

#### 5.4.2. Limitations of Our Work

Although the work of this paper has achieved certain performance goals in tracking key petrochemical equipment, it has not achieved overwhelming results in terms of relevant indicators. Through the analysis of algorithm modules and experimental results in this work, our algorithm has the following aspects to be improved:

1. Regarding the motion estimation model: In this work, the classic Kalman filtering is directly used in the prediction module. This modern filter is a linear model, which simply constructs a linear motion model for each component of the petrochemical equipment detection results. In the experiment, it is determined that each component of the target frame of each equipment is correlated; for example, the length and height of the target frame changes along with the camera angle of viewpoints. Therefore, in the future work, the addition of a better motion estimation model in the prediction stage of the algorithm will be the focus of research.
2. Regarding the tracking speed: As far as the speed of the multi-object tracking algorithm is concerned, the tracking speed of the proposed algorithm is slower than that of other algorithms such as SORT, DeepSORT, FairMOT and CenterTracker. Besides this, it should be noted that when the acquisition speed and image resolution in the mobile cameras are improved, the processing speed of our model decreases. The change in WLAN and the data server and computing server, e.g., the videos are stored in a different server while our model in the computing server has to access the videos through a different WLAN, also affects the real-time visualization performance of our model in the whole system. Based on these considerations, the future improvement direction is to make the detection model lightweight and balance the accuracy and speed of the model by deleting redundant modules in the network structure. For the appearance of feature distance and ReID network module, we will design a simplified and effective appearance feature extraction backbone to ensure the quality of appearance features and reduce the dimension of appearance features to speed up the appearance feature recovery module. In addition, we will explore the some quantization methods in reviewing [43], weight-quantized SqueezeNet [44], and sparse techniques such as sparse Yolov2 [45] and automatic sparse connectivity learning [46] to decrease the memory usage. The above operations will further benefit the speed of the multi-object tracking algorithm to balance the accuracy and speed.

## 6. Conclusions

In this paper, aiming at resolving the tracking problem of key equipment in the moving camera viewpoint in petrochemical enterprise on-site working scene, a multi-object tracking data set of petrochemical equipment is constructed, and a multi-object tracking algorithm is proposed based on the strategy of “tracking-by-detection”. Aiming at the problem that the target scale of petrochemical equipment changes greatly and the detection performance of small targets is poor under the moving camera perspective, an improved Yolov7 equipment detector is proposed by incorporating channel and spatial attention mechanism and small target detection layer to improve the detection accuracy of key equipment under different sizes. In addition, multi-scale weighted fusion of features of different scales is used to solve the problem of large equipment scale change. To solve the problem of lost tracking target caused by complex background interference and occlusion, this paper proposes a Siamese network with attention mechanism to extract appearance features and strengthen the ability to distinguish different equipment. In order to speed up data association, this paper also introduces a traditional nonparametric local texture feature descriptor LBPH, depth metric learning feature, and location feature to perform a hybrid similarity matching for key equipment, and sets reasonable thresholds to exclude significantly dissimilar key equipment pairs. The experiment on the petrochemical equipment tracking data set shows that the tracking accuracy and precision of the proposed algorithm reach 86.4% and 78.8% under the moving perspective and reduce the identity switching frequency of key equipment to a certain extent, which can basically meet the tracking stability requirements of multiple key equipment in real petrochemical working scenes.

## Figures and Tables

**Table 1:** Details of the self-built videos captured in different petrochemical plants.

Tracking Task	Resolution	Frames	Objects to Be Tracked	Number of Labeling
No1	540 × 960	308	384	1224
No2	1280 × 720	366	467	1528
No3	1920 × 1080	472	540	1626
No4	2800 × 1500	386	435	2006
No5	2800 × 1500	542	628	2568

**Table 2:** Quantitative comparison measured by the common metric mAP in percentage with IoU = 0.5 and 0.75 of detection precision on different petrochemical equipment. “*” indicates the difference in mean of the proposed model in *t*-test at 95% significance level.

Method	*mAP*50 (All)	*mAP*75 (All)	Screw Pump	Cylinder	Centrifugal Pump	Sphere	Heat Interchanger
Faster RCNN [14]	95.9 *	91.2 *	94.3 *	98.4 *	97.1 *	95.4 *	93.8 *
SSD [30]	96.2 *	93.8 *	95.2 *	98.1 *	96.6 *	95.2 *	94.9 *
RetinaNet [31]	96.3 *	93.5 *	95.5 *	99.2	96.1 *	96.8 *	94.2 *
EfficientDet [32]	96.2 *	94.8 *	93.8 *	98.4 *	96.6 *	97.2 *	96.3 *
Yolov5x [29]	97.4 *	95.6 *	96.4	99.5	98.1 *	97.5	97.0 *
Yolov7 [8]	97.6	96.1 *	96.5	99.5	98.3 *	97.4	97.2
Proposed	97.9	96.5	96.7	99.5	98.7	97.6	97.3

**Table 3:** Ablation study of tracking performance on the test set of our chemical equipment data set.

Model	Yolov7-CA	SiameseNet-CA	Hybrid Similarity	MOTP (↑)	MOTA (↑)	MT (↑)	ML (↓)	IDSw (↓)	FPS (↑)
DeepSORT	-	-	-	85.6	77.8	64	35	186	27.5
V1	√	-	-	86.0	78.2	66	34	173	26.8
V2	-	√	-	85.9	78.1	65	35	170	26.7
V3	-	-	√	85.8	78.1	64	34	172	27.3
Proposed	√	√	√	86.4	78.8	67	33	160	25.2

**Table 4:** Tracking performance of compared algorithms on the test set of our chemical equipment data set.

Model	MOTP (↑)	MOTA (↑)	MT (↑)	ML (↓)	IDSw (↓)	FPS (↑)
SORT [2]	82.7	75.7	59	41	231	32.6
DeepSORT [3]	85.6	77.8	64	35	186	27.5
ByteTrack [4]	86.3	78.8	68	34	162	24.6
FairMOT [42]	85.7	78.0	67	34	162	25.8
CenterTracker [6]	85.2	77.3	62	43	178	28.4
Proposed	86.4	78.8	67	33	160	25.2

## Data Availability

The processed data are available upon request to all the corresponding authors.
